# Diagnostic and Prognostic Value of Arterial Blood Gas and Electrolyte Analyses in Heart Failure

**DOI:** 10.31083/RCM47958

**Published:** 2026-03-19

**Authors:** Nardi Tetaj, Andrea Segreti, Francesco Piccirillo, Michele Pelullo, Simone Pasquale Crispino, Martina Ciancio, Gian Paolo Ussia, Francesco Grigioni

**Affiliations:** ^1^Cardiology Unit, Fondazione Policlinico Universitario Campus Bio-Medico, 00128 Roma, Italy; ^2^Research Unit of Cardiovascular Science, Department of Medicine and Surgery, Università Campus Bio-Medico di Roma, 00128 Roma, Italy

**Keywords:** acute heart failure, chronic heart failure, arterial blood gas, lactate, electrolyte imbalance

## Abstract

Heart failure (HF) is a multifaceted clinical syndrome that frequently precipitates disturbances in perfusion, ventilation, and metabolic regulation, all of which are rapidly detectable through arterial blood gas (ABG) analysis. Meanwhile, clinical markers such as lactate, arterial pH, arterial partial pressure of carbon dioxide (PaCO_2_), arterial partial pressure of oxygen (PaO_2_), bicarbonate, and electrolyte concentrations provide dynamic insight into the pathophysiologic status of patients and can serve as early indicators of decompensation. This review evaluates the clinical significance of key ABG and electrolyte parameters in both acute and chronic HF, emphasizing the prognostic value of the analyses, contribution to risk stratification, and utility in guiding therapy. In acute HF and cardiogenic shock, hyperlactatemia and acidosis are associated with increased mortality and the need for hemodynamic or ventilatory support. Furthermore, electrolyte abnormalities, particularly those involving sodium and potassium, are common and driven by neurohormonal activation, pharmacological therapies, and volume shifts. Therefore, integrating ABG and electrolyte monitoring into routine HF management can enhance diagnostic precision and support timely, targeted interventions. This narrative review synthesizes current evidence and proposes a practical framework for interpreting ABG results in the context of contemporary HF care.

## 1. Introduction

Heart failure (HF) is a complex clinical syndrome characterized by structural or functional cardiac impairment, leading to elevated intracardiac pressures and/or reduced cardiac output, either at rest or during exertion [1]. This hemodynamic dysfunction manifests with cardinal symptoms such as breathlessness, fatigue, and ankle swelling, often accompanied by signs including elevated jugular venous pressure, pulmonary rales, and peripheral edema. The diagnosis of chronic HF requires the presence of symptoms and/or signs of HF and objective evidence of cardiac dysfunction. The etiology of HF is heterogeneous and often multifactorial, with ischemic heart disease and hypertension as predominant causes globally [1, 2]. HF is an emerging public health priority, driven primarily by demographic aging and the rising incidence of risk factors, including hypertension, obesity, and diabetes. The estimated lifetime risk of developing HF is 20–25%, with one in four individuals likely to experience the condition during their lifetime [3, 4]. Despite therapeutic advances, HF continues to be associated with high mortality with 1-year and 5-year mortality rates approximately of 20% and up to 50%, respectively. Among older patients (≥65 years) hospitalized for HF, the 1-year post-discharge mortality approaches 35%, underscoring the urgent need for improved risk stratification tools [5, 6, 7, 8].

Arterial blood gas (ABG) offers rapid insight into the respiratory, metabolic, and perfusion status of patients with HF. Parameters such as lactate, pH, partial pressure of carbon dioxide (PaCO_2_) and oxygen (PaO_2_), the PaO_2_/FiO_2_ ratio, bicarbonate concentration (HCO_3_^–^), and electrolytes provide rapid insights into tissue perfusion, acid-base status, and cardiorenal-respiratory interplay [9, 10].

Due to its rapid turnaround, bedside availability, and favorable safety profile, ABG analysis is widely used in both emergency and inpatient settings to support immediate clinical decision-making. Emerging evidence suggests that specific ABG variables at presentation are significantly associated with critical clinical outcomes in acute HF, including mortality, need for mechanical ventilation, ICU admission, and prolonged hospitalization [11, 12].

This narrative review explores the diagnostic and prognostic utility of ABG including electrolytes in patients with HF, focusing on their clinical relevance, pathophysiological implications, and role in contemporary HF management.

## 2. Lactate: Marker of Tissue Hypoperfusion

Lactate is a key biomarker of anaerobic metabolism and tissue hypoperfusion. Under conditions of impaired oxygen delivery, pyruvate generated from glycolysis is reduced to lactate by L-lactate dehydrogenase. Lactate is then transported to the liver, where it is oxidized back to pyruvate, and undergoes gluconeogenesis or enters the Krebs cycle as acetyl-CoA [13]. Approximately 70–75% of circulating lactate is metabolized by the liver; the kidneys contribute the remaining clearance. Hyperlactatemia (≥2 mmol/L) is frequently observed in conditions such as sepsis, shock, intense exercise, and seizures [14].

During shock, lactate becomes a primary energy substrate for the heart. Hyperlactatemia reflects a stress response characterized by increased metabolic rate, sympathetic activation, accelerated glycolysis, and altered bioenergetic pathways [15]. While mild lactate elevations may occur in chronic HF, levels are often significantly higher in acute HF (AHF). The underlying mechanism can differ between the conditions. In chronic HF, hyperlactatemia is thought to result from increased glycolysis due to chronic metabolic dysregulation, sustained myocardial injury, and persistent sympathetic nervous system activation, which together enhance lactate production and efflux from cells [16].

In contrast, acute HF is typically associated with systemic hypotension, hypoxia, and hypoperfusion, which shift metabolism toward anaerobic pathways and markedly increase lactate levels. In advanced stages, multiorgan dysfunction, including hepatic and renal impairment, further limits lactate clearance, exacerbating hyperlactatemia [17].

**Fig. 1.**
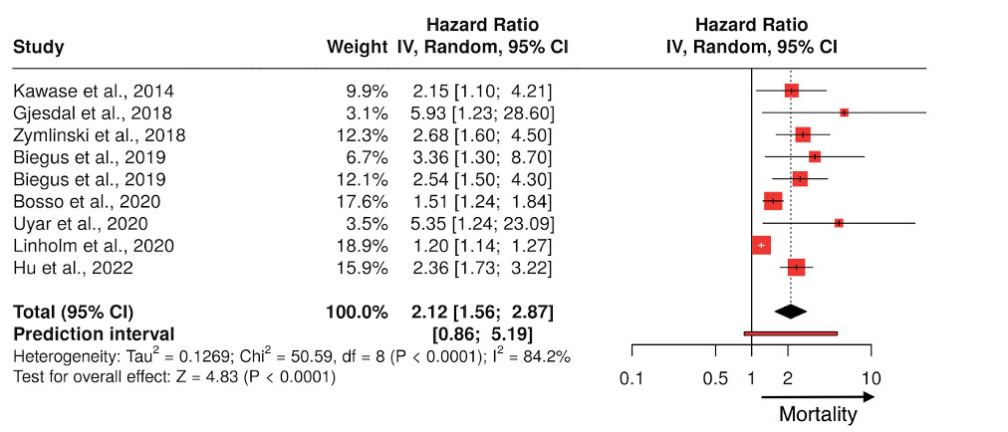
**Forest plot summarizing the clinical studies investigating lactate as a prognostic marker in patients with heart failure**. IV, intravenour.

Multiple studies, as shown in Table 1 (Ref. [18, 19, 20, 21, 22, 23, 24, 25, 26, 27]) and Fig. 1, have demonstrated the prognostic value of elevated lactate levels in AHF, even in the absence of overt clinical signs of hypoperfusion [18]. High lactate correlates with in-hospital mortality, the need for circulatory support and ICU admission [19]. Conversely, normalization of lactate levels, or evidence of lactate clearance, is a favorable prognostic sign. Lactate thus serves as both a prognostic indicator and a therapeutic target in the acute management of HF [20, 28, 29]. In cardiogenic shock, lactate is integral to the Society for Cardiovascaular Angiography and Interventions (SCAI) staging system, helping identify patients who may benefit from advanced therapies [30].

**Table 1.** **Summary of clinical studies investigating lactate as a prognostic marker in patients with heart failure**.

Study (year)	Type of study	Sample size	HF type	Lactate threshold	Outcomes	OR (95% CI)	*p*-value
Kawase *et al*. (2015) [19]	Retrospective single-center observational study	754	Acute HF in ICU	>3.2 mmol/L	In-hospital all-cause death	2.14 (1.10–4.21)	0.03
Gjesdal *et al*. (2018) [22]	Retrospective single-center observational study	1260	Patients with AMI underwent PCI and with signs of mild to moderate heart failure (Killip class II–III)	≥2.5 mmol/L	30-day mortality	5.94 (1.23–28.64)	<0.05
Zymliński *et al*. (2018) [18]	Retrospective single-center observational study	237	Patients with AHF without overt clinical evidence of peripheral hypoperfusion	≥2 mmol/L	All-cause 1-year mortality	2.7 (1.6–4.5)	<0.0001
Biegus *et al*. (2019) [23]	Retrospective single-center observational study	89	Hospitalized patients with AHF	>2 mmol/L	One-year mortality	3.4 (1.3–8.7)	0.009
Biegus *et al*. (2019) [24]	Prospective single-center observational study	222	Hospitalized patients with Acute HF	≥2 mmol/L	Persistent hyperlactataemia within the first 24 h of hospitalization is a predictor of a worse outcome in AHF and is related to higher rates of in-hospital adverse events and one-year mortality	2.5 (1.5–4.3)	<0.001
Bosso *et al*. (2021) [25]	Prospective single-center observational study	96	AHF presenting to Emergency	≥2 mmol/L (24-hour time‐weighted lactate)	In-hospital composite outcome (need for ICU admission, LOS >7 days and in-hospital mortality)	1.51 (1.24–1.84)	<0.001
Uyar *et al*. (2020) [26]	Prospective single-center observational study	85	AHF admitted to the hospital	≥2 mmol/L	Composite of cardiovascular death and HF hospitalizations at 6 months	5.35 (1.243–23.093)	0.024
Lindholm *et al*. (2020) [27]	Post hoc analysis of CardShock study	217	AMI-CS	≥2 mmol/L	30-day all-cause mortality	1.20 (1.14–1.27)	<0.0001
Marbach *et al*. (2022) [20]	Post-hoc analysis of the DOREMI trial	142	All-cause cardiogenic shock (SCAI stages B–E)	Lactate clearance at 24 hours	In-hospital survival	5.44 (2.14–13.8)	< 0.01
Hu *et al*. (2018) [21]	Retrospective single-center observational study	7558	Acute HF admitted in ICU	2.3–4.3 mmol/L	In-hospital all-cause mortality was gradually increased with lactic acid levels increasing	2.36 (1.73–3.22)	< 0.05

Abbreviations: AMI, acute myocardial infarction; PCI, percutaneous coronary intervention; HF, heart failure; SCAI, Society for Cardiovascular Angiography and Interventions; LOS, length of stay; AHF, acute HF; OR, odds ratio; CI, confidence interval.

The lactate/albumin (L/A) ratio has been linked with poorer outcomes in adults suffering from a variety of conditions such as sepsis, trauma and heart failure. Some studies suggest that the L/A ratio may correlate with poor prognosis [31, 32].

Furthermore, studies have shown that lactate accumulation in acute HF is closely related to cardiac index, with mixed venous oxygen saturation, heart rate, and systemic vascular resistance emerging as the strongest determinants [33].

In clinical practice, a serum lactate >2–4 mmol/L is often regarded as a red flag. Serial lactate monitoring is also valuable, as trends can reflect clinical trajectory and treatment response. Lactate is an easily obtainable ABG parameter that supports risk stratification. Elevated levels should prompt clinicians to consider aggressive hemodynamic and respiratory support, given the strong association with increased mortality. For frontline providers, an elevated lactate in the setting of HF should raise suspicion for cardiogenic shock or peripheral hypoperfusion and prompt urgent diagnostic and therapeutic evaluation.

From a practical standpoint, the interpretation of lactate in HF should move beyond a single admission value and incorporate both the underlying HF phenotype and serial trends over time. Patients with cardiogenic shock, mixed cardiogenic–septic shock, or advanced chronic HF with multiorgan dysfunction may all exhibit elevated lactate, but for different pathophysiological reasons, including impaired tissue perfusion, adrenergic stimulation, hepatic and renal dysfunction, or β-agonist therapy. In this context, lactate clearance, typically assessed over the first 2–4 hours and again at 12–24 hours, functions less as a stand-alone therapeutic target and more as a dynamic marker of global response to therapy. Conversely, absent or minimal lactate clearance should prompt reassessment for ongoing hypoperfusion, inadequate cardiac output, unrecognized infection, or drug-related contributors, and may support early escalation to pharmacological, mechanical circulatory or respiratory support. Future HF-specific studies stratified by phenotype, comorbidities (e.g., chronic kidney or liver disease, diabetes, COPD), and treatment strategy are needed to validate lactate kinetics as a formal resuscitation endpoint in this population.

## 3. Arterial pH and Bicarbonate Buffer: Acid-Base Interpretation

Arterial pH reflects the balance between the respiratory (PaCO_2_) and metabolic (HCO_3_^–^) systems, components of acid-base homeostasis. In red blood cells, carbon dioxide (CO_2_) combines with water under the action of carbonic anhydrase to form carbonic acid, which rapidly dissociates into bicarbonate (HCO_3_^–^) and hydrogen ions (H^+^). CO_2_ crosses cell membranes via simple diffusion, dissolves in the blood, and is primarily eliminated through pulmonary exhalation. This process is modulated by the rate and depth of respiration. The level of bicarbonate in the blood is controlled through the renal system, where it is filtered and then reabsorbed in the proximal convoluted tubule [34].

**Fig. 2.**
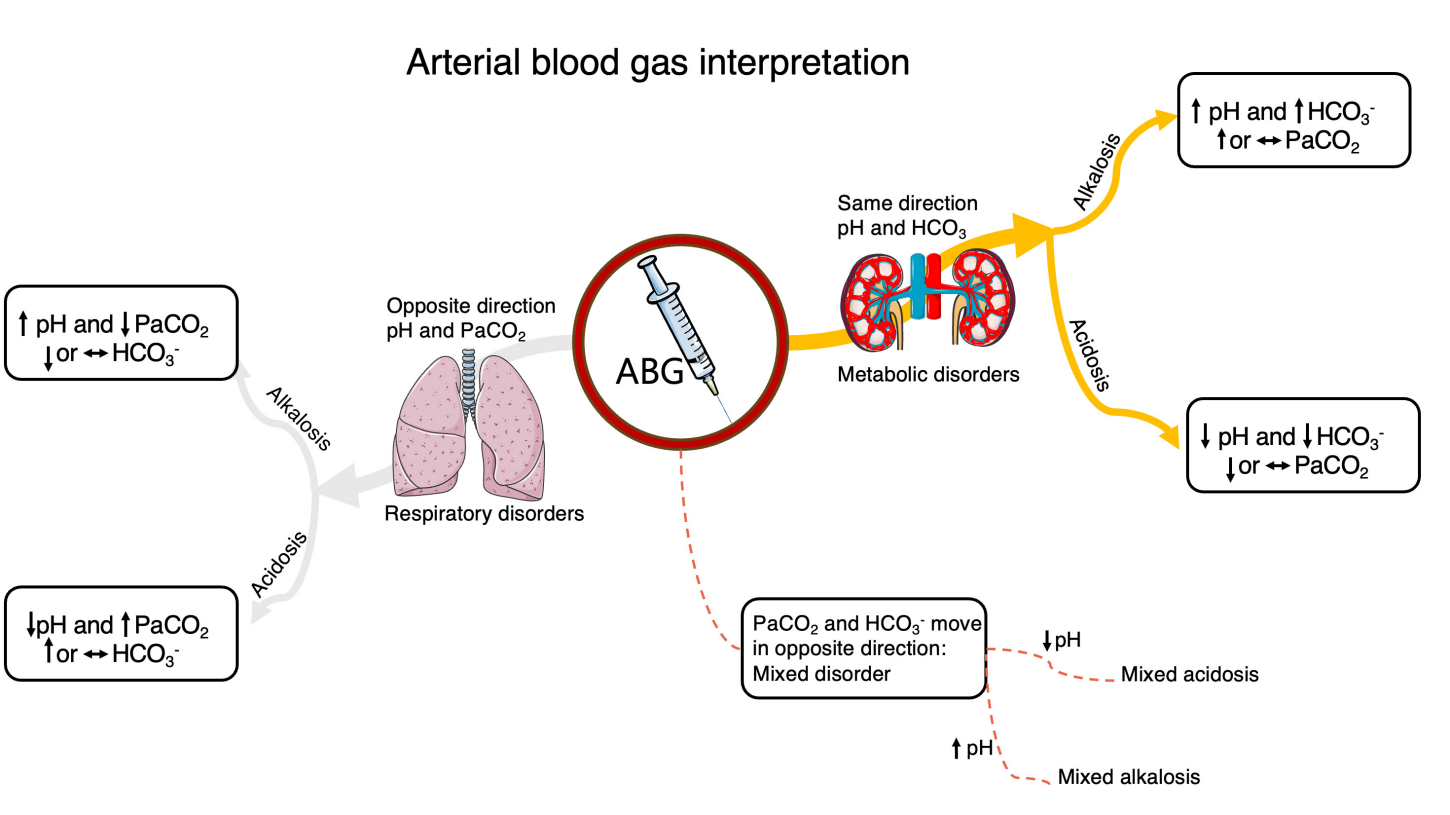
**An example of ABG interpretation**. Abbreviations: ABG, arterial blood gas; PaO_2_, arterial partial pressure of oxygen; PaCO_2_, arterial partial pressure of carbon dioxide; HCO_3_, carbonate ion; ↑, increase; ↓, decrease; ↔, in range.

In a normal ABG analysis, HCO_3_^–^ and PaCO_2_ typically shift in the same direction as part of a compensatory mechanism (Fig. 2); however, renal compensation is generally slower than respiratory compensation. When both pH and HCO_3_^–^ change in the same directions, a primary metabolic disorder is likely. Conversely, when pH and PaCO_2_ move in opposite direction, the primary disorder is respiratory [35]. In contrast, a mixed disorder is characterized by HCO_3_^–^ and PaCO_2_ moving in opposite directions, a pattern that is not expected in isolated disturbances, and pH may be either normal or abnormal.

In HF, acidemia (pH <7.35) generally indicates a state of lactic acid accumulation secondary to tissue hypoperfusion (metabolic acidosis), or from respiratory acidosis caused by a state of hypercapnia due to ventilatory failure (respiratory acidosis) [36]. Conversely, respiratory alkalosis may arise from hyperventilation, often triggered by pulmonary congestion and the activation of J-receptors due to increased lung water. Metabolic alkalosis is also frequently observed, particularly in the context of high-dose diuretic therapy [37].

Also, lactate can acutely stimulate carotid chemoreceptors, much like hypoxia and is able to stimulate carotid body sensory activity in the absence of other hypoxic signals, increasing respiratory rate [38].

Several studies have identified acidosis at admission as a predictor of mortality in acute HF [37, 39]; however, little evidence has yielded conflicting results [40]. These discrepancies may be attributed to substantial differences in the characteristics of the study populations. A large multinational registry of 1982 AHF patients (KorHF Registry) stratified patients by admission pH. Acidosis was present in roughly 19% on arrival, primarily metabolic or mixed-type acidosis. In adjusted Cox analysis, admission acidosis emerged as an independent predictor of mortality (hazard ratio 1.9, 95% CI 1.27–2.93). The largest group had respiratory alkalosis, whereas only 7% had metabolic alkalosis. Notably, alkalosis was not associated with increased mortality in that cohort [9].

An arterial pH <7.35 in the setting of AHF should heighten clinical concern, which could reflect worse perfusion and possible circulatory shock. Acidosis may prompt more aggressive management: for example, initiating inotropes or mechanical circulatory support in cardiogenic shock, or using ventilation (noninvasive or invasive) if respiratory acidosis is present. Mild alkalosis, often from hyperventilation due to hypoxia or pain, is common and in itself is not linked to worse mortality [41]. Thus, the presence of acidosis is the key pH-related warning sign. Identifying acidosis early allows clinicians to address its reversible causes (e.g., improve perfusion, treat underlying ischemia or arrhythmia, optimize ventilation) and potentially improve outcomes.

Acid-base status also exerts a powerful influence on electrolyte distribution, particularly for potassium and chloride, and this interaction is frequently encountered in HF. In metabolic or respiratory acidosis, hydrogen ions move into cells and potassium shifts to the extracellular space, leading to hyperkalemia even when total body potassium is normal or reduced. Conversely, acute respiratory or metabolic alkalosis promotes intracellular potassium uptake, predisposing to hypokalemia and increasing the risk of ventricular arrhythmias in patients already vulnerable due to structural heart disease. Chloride, together with sodium and bicarbonate, is a key determinant of the strong ion difference: hyperchloremia from chloride-rich fluids can narrow the strong ion difference and drive a non-anion gap metabolic acidosis, whereas chloride loss from vomiting or loop and thiazide diuretics widens it and contributes to metabolic alkalosis. In HF, these mechanisms often coexist—for example, a decompensated patient with hypercapnic acidosis on non-invasive ventilation may develop rising potassium levels despite stable renal function, while another on high-dose loop diuretics may present with metabolic alkalosis, hypochloremia, and hypokalemia. Recognizing these linked patterns on ABG and electrolyte panels is essential for targeted correction and for avoiding oversimplified interpretations such as attributing all abnormalities solely to “renal failure” or “diuretic therapy”.

### 3.1 Arterial Oxygenation, P/F Ratio and Venous Oxygenation

The arterial partial pressure of oxygen (PaO_2_) in arterial blood is a key component of ABG analysis. The PaO_2_/FiO_2_ ratio (arterial oxygen partial pressure divided by the inspired oxygen fraction) is a well-established index of oxygen perfusion. It is used to characterize acute respiratory distress syndrome (ARDS) severity as per the Berlin definition, but it is equally applicable to cardiogenic pulmonary edema and correlates with the need for ventilatory support and ICU care [42, 43].

In acute HF, the rapid accumulation of fluid within the interstitial and alveolar spaces due to elevated cardiac filling pressure, leading to acute cardiogenic pulmonary edema. Edema reduces pulmonary compliance, promotes alveolar and small airway collapse, gas exchange can be severely impaired, leading to hypoxemia and significant dyspnea. A low PaO_2_/FiO_2_ ratio in AHF indicates significant intrapulmonary shunting or diffusion impairment due to fluid-filled alveoli. The early application of positive end-expiratory pressure (PEEP) keeps the airway open, counteracting alveolar collapse and improving gas exchange [44].

**Fig. 3.**
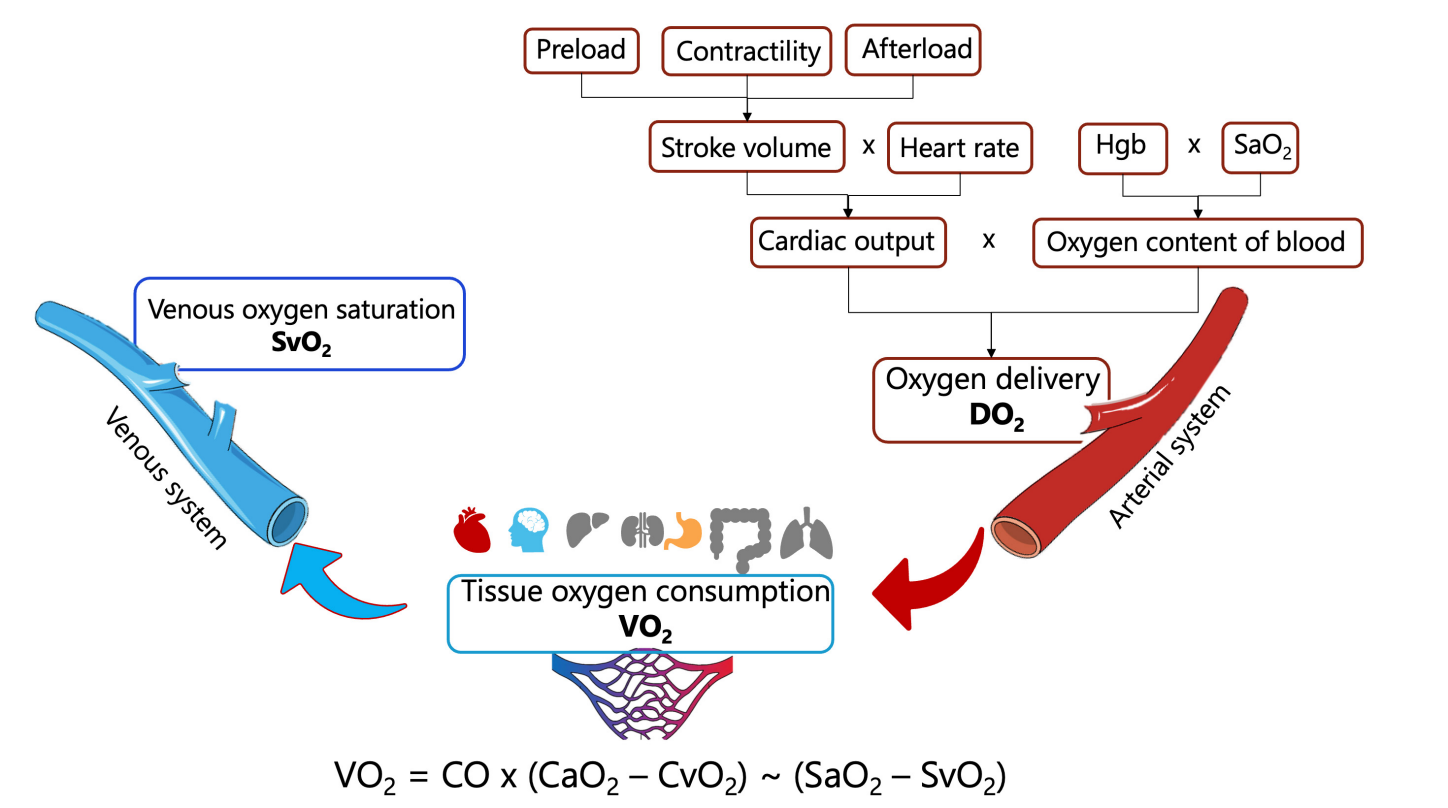
**Determinants of systemic oxygen delivery and tissue oxygen consumption**. Abbreviations: DO_2_, systemic oxygen delivery; VO_2_, tissue oxygen consumption; SaO_2_, arterial oxygen saturation; SvO_2_, venous oxygen saturation; Hgb, hemoglobin; CO, cardiac output; CaO_2_, arterial oxygen content of blood; CvO_2_, venous oxygen content of blood.

Oxygen delivery (DO_2_) is directly proportional to hemoglobin concentration, arterial oxygen saturation (SaO_2_), and cardiac output (Fig. 3). Cardiac output, in turn, is determined by heart rate and stroke volume. The primary determinants of stroke volume include preload, afterload, and myocardial contractility. Reduction in preload or contractility typically diminishes stroke volume, whereas elevated afterload may impede ventricular ejection, thereby reducing stroke volume. A comprehensive understanding of these hemodynamic relationships is essential for the assessment and management of cardiovascular conditions such as heart failure and circulatory shock [45].

Central venous oxygen saturation (ScvO_2_) represents the percentage of hemoglobin saturated with oxygen in venous blood in the right heart via a central venous catheter. It serves as a surrogate marker for the global balance between supply (DO_2_) and tissue oxygen consumption (VO_2_). The total rate of DO_2_ is usually around 15 mL/kg/min, and the normal range for VO_2_ is approximately 3.5–4.0 mL O_2_/kg/min relative to body mass. Under physiological conditions, ScvO_2_ values are between 70 and 75%. A decrease in ScvO_2_ suggests either increased oxygen consumption (VO_2_) or DO_2_, as in cardiogenic shock [46].

Patients with chronic HF may be adapted to a low venous oxygen saturation (SvO_2_) due to chronic tissue hypoxia. An acute drop in SvO_2_ is an indication of cardiac dysfunction. On the other hand, SvO_2_ improving following cardiopulmonary resuscitation is a marker for the return of spontaneous circulation [46, 47]. Thus, in such settings, it is useful to monitor SvO_2_ [48]. Furthermore, the Surviving Sepsis Campaign guidelines recommend the use of ScvO_2_ or mixed venous oxygen saturation to assess the balance of tissue oxygen delivery and consumption in sepsis [49].

In cardiogenic shock, the veno-arterial difference in partial pressure of carbon dioxide (PCO_2_ gap or ΔPCO_2_) reflects the adequacy of cardiac output relative to metabolic CO_2_ production. Small cardiogenic shock (CS) studies suggest that higher admission ΔPCO_2_ and failure of ΔPCO_2_ to decline within 12–24 h are associated with worse outcomes, whereas decreasing values track successful resuscitation; however, a CS-specific threshold has not been validated, and ΔPCO_2_ should be interpreted alongside lactate, ScvO_2_/SvO_2_, cardiac index, and echocardiography. Derived indices such as ΔPCO_2_/Ca–vO_2_ may indicate anaerobic metabolism but lack CS-specific cut-offs and are vulnerable to confounding (e.g., anemia, changes in ventilation, temperature, or CO_2_ production).

In VA-ECMO, ABG interpretation becomes more complex because measured PaO_2_ and PaCO_2_ depend on cannulation configuration, native cardiac output, circuit flow, and the degree of mixing between oxygenated extracorporeal blood and desaturated native cardiac output. Samples drawn from the right radial artery, femoral artery, or post-oxygenator line may yield substantially different results, particularly in the presence of “north-south” (Harlequin) syndrome with preserved left ventricular ejection and severe pulmonary dysfunction. In this setting, a normal PaO_2_ in a post-oxygenator sample can coexist with cerebral or myocardial hypoxia, while extracorporeal CO_2_ removal may mask ongoing tissue hypoperfusion if lactate and ΔPCO_2_ trends are not assessed in conjunction with circuit parameters and clinical examination. For these reasons, ABG results in VA-ECMO patients should be interpreted in a site-specific manner and always integrated with echocardiography, invasive hemodynamics, and regional perfusion markers rather than used in isolation [50, 51, 52].

ABG-derived indices such as lactate, PaO_2_/FiO_2_, PaCO_2_, and ScvO_2_ have the potential to complement existing HF and cardiogenic shock risk scores. Lactate is already incorporated into several shock staging systems and reflects the severity of systemic hypoperfusion, while PaO_2_/FiO_2_ captures the burden of respiratory failure and pulmonary congestion and ScvO_2_ provides a dynamic estimate of the balance between oxygen delivery and consumption. Integrating these parameters into multiparametric scores that also include clinical signs, biomarkers, and imaging findings could improve discrimination and reclassification for key outcomes such as the need for mechanical circulatory support, ICU admission, or short-term mortality. In practice, serial changes in lactate, PaO_2_/FiO_2_ and ScvO_2_ under therapy may be more informative than single measurements at admission, helping to identify patients with a “failing trajectory” who warrant early escalation of support despite apparently stable vital signs.

### 3.2 Electrolytes Implications in Heart Failure

Chronic HF is characterized by progressive neurohormonal and hemodynamic dysregulation that triggers compensatory mechanisms aimed at maintaining perfusion. Among these, sympathetic nervous system hyperactivity initially supports circulatory homeostasis but eventually leads to β-adrenergic receptor downregulation and catecholamine depletion—further impairing myocardial function and reducing inotropic reserve [53, 54]. This pathophysiologic rationale underpins the central role of β-blockers as one of the four foundational pillars of heart failure therapy in patients with reduced ejection fraction (HFrEF), as outlined by current ESC guidelines [55, 56].

**Fig. 4.**
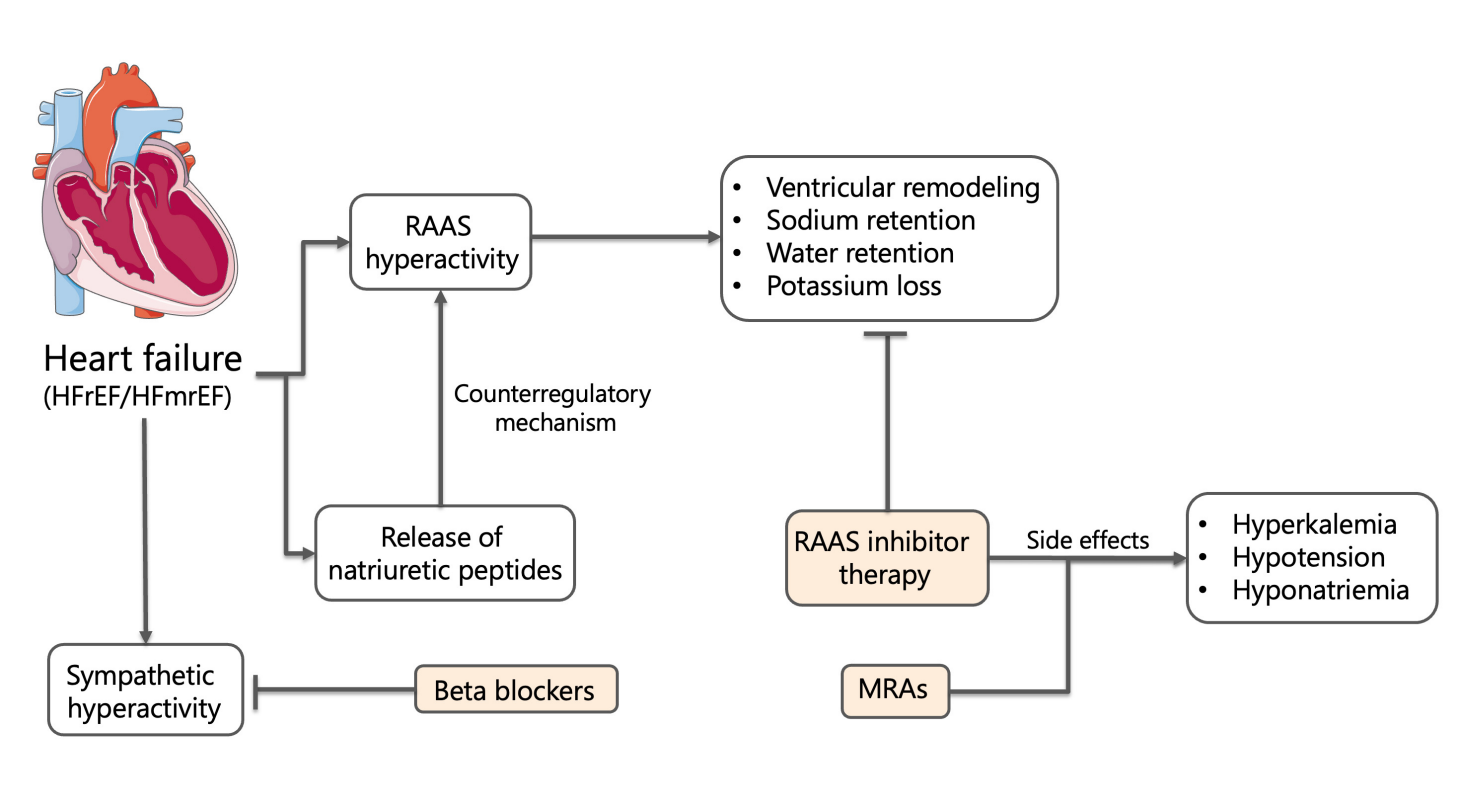
**Neurohormonal activation and pharmacological modulation in heart failure**. Abbreviations: HFrEF, heart failure with reduced ejection fraction (≤40%); HFmrEF, heart failure with mildly reduced ejection fraction (41–49%); RAAS, renin-angiotensin-aldosterone system; MRAs, mineralocorticoid receptor antagonists.

In parallel, activation of the renin-angiotensin-aldosterone system (RAAS) constitutes a cornerstone maladaptive response in chronic HF. Reduced cardiac output and renal perfusion stimulate renin release from juxtaglomerular cells, initiating a cascade culminating in angiotensin II and aldosterone secretion. Angiotensin II promotes vasoconstriction and adverse ventricular remodeling, while aldosterone induces renal sodium and water retention, potassium excretion, and myocardial fibrosis. Antidiuretic hormone (ADH) release is concurrently stimulated, compounding sodium-free water retention and contributing to dilutional hyponatremia and volume overload. Although natriuretic peptides such as BNP and ANP are released in response to myocardial stretch, their compensatory effects are often overwhelmed in advanced disease stages [57]. The pathologic consequences of sustained RAAS activation are central to the progression of heart failure, as shown in Fig. 4. Angiotensin II and aldosterone drive myocardial fibrosis, hypertrophy, endothelial dysfunction, and adverse remodeling—amplifying both systolic and diastolic impairment [58, 59].

These neurohormonal and hemodynamic adaptations not only modulate sodium and water balance but also generate characteristic acid-base and electrolyte patterns that are readily captured by ABG analysis. For example, RAAS activation and aldosterone excess favor renal potassium loss and metabolic alkalosis, particularly when combined with loop or thiazide diuretics, whereas advanced renal dysfunction, RAAS inhibition, and acidosis shift the balance toward hyperkalemia and mixed metabolic disturbances. Thus, the electrolyte profile of a patient with HF is best interpreted in parallel with ABG-derived pH, PaCO_2_, and bicarbonate, viewing both as different facets of the same pathophysiological process rather than isolated laboratory domains.

The therapeutic goal in HF is to reduce mortality, mitigate symptoms, prevent hospitalization, and improve quality of life. ESC guidelines endorse four classes of disease-modifying therapies for HFrEF and HF mildly reduced ejection fraction (HFmrEF): (1) RAAS inhibitors, including angiotensin converting enzyme (ACE) inhibitors, angiotensin receptor blockers (ARBs), and angiotensin receptor-neprilysin inhibitors (ARNIs); (2) β-blockers; (3) mineralocorticoid receptor antagonists (MRAs); and (4) sodium-glucose cotransporter-2 inhibitors (SGLT2i). In patients with congestion, diuretics provide symptomatic relief and facilitate reverse remodeling. In selected cases, in valvular heart disease, structural interventions, such as transcatheter or surgical valve repair, may offer additional benefit [2].

While RAAS inhibition improves outcomes, it may provoke clinically significant electrolyte disturbances. Suppressed aldosterone activity can lead to hyponatremia, hypotension, and volume depletion—particularly in patients on loop diuretics or sodium-restricted diets [60]. Simultaneously, impaired potassium excretion predisposes to hyperkalemia, especially in individuals with chronic kidney disease (CKD) or those receiving potassium-sparing agents [61]. Thus, the therapeutic target needed in HF patients is often not reached because of these side-effects. Management of these side-effects is pivotal to maximize the use of renin-angiotensin-aldosterone system inhibitors (RAASi) in HFrEF, particularly in high-risk patients.

Furthermore, SGLT2i, the latest foundational therapy in HFrEF, exert its primary action by promoting glucosuria and natriuresis in the proximal renal tubule. These agents offer a unique hemodynamic and electrolyte-sparing profile, although, due to osmotic diuresis, they can occasionally lead to hypotension and mild hyponatriemia, particularly in elderly or volume-depleted patients [62].

## 4. ABG and Electrolyte Monitoring

Beyond its traditional application in acid-base analysis, ABG testing now serves as a rapid, point-of-care tool for assessing electrolytes and guiding real-time management in critically ill heart failure patients. ABG analyzers provide concurrent measurements of sodium, potassium, chloride, bicarbonate, and ionized calcium—electrolytes integral to maintaining cardiac electrophysiology, volume status, and acid-base balance.

The advantage of ABG-based electrolyte assessment in HF is the ability to interpret these values in the immediate context of pH, PaCO_2_, bicarbonate, and lactate. For example, a mild elevation in potassium will carry different clinical implications in a patient with metabolic alkalosis and total body potassium depletion than in one with metabolic or respiratory acidosis on the verge of malignant arrhythmias. Similarly, the combination of rising chloride and falling bicarbonate on ABG may uncover iatrogenic hyperchloremic acidosis from saline administration, whereas low chloride with elevated bicarbonate suggests diuretic-induced metabolic alkalosis and neurohormonal activation. This integrated, physiology-based interpretation is more informative than viewing electrolyte values in isolation from the concurrent ABG profile.

Sodium is the predominant extracellular cation, essential for maintaining osmolality, intravascular volume and hemodynamic stability. It also contributes to acid-base balance through the calculation of the anion gap: AG = [Na^+^] – ([Cl^–^] + [HCO_3_^–^]).

An elevated anion gap suggests the accumulation of unmeasured anions (e.g., lactate, ketones), characteristic of high anion gap metabolic acidosis [63]. In Chronic HF (CHF), hyponatremia is independently associated with poor prognosis, including increased mortality and rehospitalization rates [64]. Sodium derangements may be further exacerbated by overly aggressive sodium restriction, highlighting the need for individualized dietary and pharmacologic strategies [60].

Potassium is the primary intracellular cation and is crucial in maintaining cellular membrane potential, myocardial conduction, and skeletal muscle function. Approximately 98% of total body potassium is located intracellularly, with only a small fraction circulating in the extracellular space. This distribution is tightly regulated by the Na^+^/K^+^ ATPase pump and influenced by acid-base status, insulin, and adrenergic activity. In acidosis, potassium shifts extracellularly in exchange for hydrogen ions, resulting in hyperkalemia; alkalosis produces the opposite effect. Importantly, extracellular potassium may not accurately reflect total body stores. Normokalemia or even hyperkalemia may mask underlying depletion [65].

Hyperkalemia (>5.5–6.0 mmol/L) and hypokalemia (<3.5 mmol/L) substantially increase the risk of malignant ventricular arrhythmias and sudden cardiac death [66]. Frequent monitoring—particularly via ABG in decompensated patients, renal dysfunction, or during RAAS inhibitor titration—is essential. Management may require potassium binders, loop diuretics, dialysis, or temporary adjustment of RAAS-modulating agents. Management of hyperkalemia and hypokalemia in heart failure is shown in Tables 2,3.

**Table 2.** **Practical management of hyperkaliemia in heart failure**.

Serum K^+^ (mmol/L)*	ECG/clinical risk	Acute management	HF-specific medium/long-term management
Mild ↑ K^+^ 5.1–5.5	Usually no ECG changes	- Confirm true hyperkalemia (exclude hemolysis; repeat K^+^, ABG, check renal function and acid–base status).	- Optimize guideline-directed HF therapy: maintain RAASi/MRA if possible; consider small dose reduction rather than withdrawal.
		- Review medications: RAASi/MRA, K^+^-sparing diuretics, NSAIDs, trimethoprim, heparin.	- Advise moderate dietary K^+^ restriction (avoid high-K^+^ foods and salt substitutes).
		- No need for emergency K^+^ lowering if asymptomatic and ECG normal.	- Intensify loop or thiazide diuretic if volume overloaded.
			- In recurrent hyperkalemia or CKD, consider chronic K^+^ binders (patiromer or sodium zirconium cyclosilicate) to allow continuation/up-titration of RAASi/MRA.
Moderate ↑ K^+^ 5.6–6.0	May be asymptomatic or show subtle ECG changes (peaked T waves); higher arrhythmic risk in HFrEF, or ischemic cardiomyopathy	- Repeat ABG and K^+^ to confirm. - 12-lead ECG and continuous monitoring if K^+^ ≥5.8–6.0 or high-risk HF profile. - If no ECG changes and hemodynamically stable: consider loop diuretic IV or PO, adjust RAASi/MRA dose, and start a K^+^ binder early. - Address triggers (dehydration, AKI, high-K^+^ diet, metabolic acidosis).	- Reassess need/dose of ACEi/ARBs/ARNIs and MRA; try to maintain life-saving drugs using K^+^ binders and diuretics rather than stopping them outright.
			- Schedule close K^+^ and creatinine monitoring (e.g., within 48–72 h after any medication change).
			- Educate patient on diet, over-the-counter drugs (NSAIDs), and sick-day rules.
Severe ↑ K^+^ ≥6.0	Very high risk of malignant arrhythmias and cardiac arrest	1. Stabilize myocardium: IV calcium gluconate or calcium chloride (according to local protocol) if ECG changes. Avoid calcium if digoxin toxicity is suspected. 2. Shift K^+^ intracellularly: - IV insulin + glucose (avoid excessive volume in HF; use concentrated dextrose and monitor glycemia). - Nebulized β2-agonist (e.g., salbutamol) if no contraindications. - IV sodium bicarbonate only if significant metabolic acidosis and appropriate volume status. 3. Remove K^+^ from body: - High-dose loop diuretic IV if euvolemic or congested and kidneys responsive. - Potassium binders (patiromer or sodium zirconium cyclosilicate) as soon as feasible. - Urgent dialysis in refractory or life-threatening hyperkalemia, especially in advanced CKD/AKI.	- After stabilization, reassess RAASi/MRA strategy: avoid permanent discontinuation if possible; restart/down-titrate under K^+^ binder and close monitoring. - Optimize diuretic regimen to prevent recurrence (consider adding thiazide in resistant edema). - Address underlying triggers (e.g., dehydration, infection, AKI, excessive K^+^ intake). - Define a target K^+^ range in HFrEF (typically 4.0–5.0 mmol/L) and individualized follow-up plan.

Abbreviations: HFrEF, heart failure with reduce ejection fraction; ECG, electrocardiogram; PO, per os; K^+^, potassium; CKD, chronic kidney disease; AKI, acute kidney injury; RAASi, renin-angiotensin-aldosterone system inhibitors; MRA, mineralocorticoid receptor antagonist; NSAIDs, nonsteroidal anti-inflammatory drugs; ACEi, angiotensin converting enzyme inhibitors; ARBs, angiotensin receptor blockers; ARNIs, angiotensin receptor-neprilysin inhibitors; *Approximate thresholds; should be interpreted in the context of local laboratory reference ranges, ECG findings, comorbidities (e.g., CKD, diabetes, COPD), and overall clinical status.

**Table 3.** **Practical management of hypokaliemia in heart failure**.

Serum K^+^ (mmol/L)*	ECG/clinical risk	Acute management	HF-specific medium/long-term management
Mild ↓ K^+^ 3.0–3.5	Often asymptomatic; increased arrhythmic risk in HFrEF, LV hypertrophy, or QT-prolonging drugs	- Confirm with repeat K^+^ and ABG. - Oral KCl supplementation if no contraindications. - Assess for concurrent hypomagnesemia and replace Mg^2+^ if low.	- Review diuretic regimen; consider reducing loop/thiazide dose or adding a K^+^-sparing agent/MRA (if not already on and no contraindication).
			- Ensure adequate RAASi/MRA and β-blocker dosing, which tend to raise or maintain K^+^.
			- Target K^+^ 4.0–5.0 mmol/L in HFrEF to reduce arrhythmic risk.
Moderate ↓ K^+^ 2.5–3.0	Increased risk of ventricular ectopy, especially with digoxin, ischemia, or LV dysfunction	- Oral KCl (divided doses) if GI tract usable and no severe symptoms. - If symptomatic, unable to take PO, or multiple risk factors for arrhythmia: slow IV KCl via peripheral or central line according to local protocols, with ECG monitoring.	- Reassess diuretic and RAASi/MRA balance: reduce loop/thiazide dose, maximize MRA if tolerated.
			- Review medications that lower K^+^ (steroids, high-dose β2-agonists, insulin regimens).
		- Correct hypomagnesemia (IV MgSO_4_ if needed).	- Arrange short-interval K^+^ checks after any change.
Severe ↓ K^+^ <2.5, or <3.0 with arrhythmias	High risk of malignant ventricular arrhythmias, especially in HFrEF and ischemic heart disease	- Continuous ECG monitoring and admission to monitored setting. - Prompt IV KCl replacement with strict rate limits and central line if high concentration is required; avoid dextrose-only fluids which can worsen hypokalemia. - Correct Mg^2+^ deficiency aggressively. - Temporarily reduce or hold digoxin and QT-prolonging drugs if feasible. - ABG monitoring to assess concomitant metabolic alkalosis or respiratory derangements.	- Once stabilized, adjust chronic therapy: lower loop/thiazide doses, up-titrate MRA/ACEi/ARBs/ARNIs if blood pressure and renal function allow. - Educate patient on maintaining adequate dietary K^+^ intake (unless contraindicated by prior hyperkalemia or CKD). - Define individualized K^+^ target and follow-up frequency based on HF phenotype, LV function, arrhythmic history, and comorbidities.

Abbreviations: *Approximate thresholds; should be interpreted in the context of local laboratory reference ranges, ECG findings, comorbidities (e.g., CKD, diabetes, COPD), and overall clinical status.

Chloride plays a pivotal role in acid-base homeostasis by its inverse relationship with bicarbonate. Hyperchloremia—often induced by excessive saline administration—can cause a non-anion gap metabolic acidosis and has been linked to worse renal outcomes [67]. Conversely, hypochloremia, may accompany metabolic alkalosis due to vomiting or diuretic use [68].

Ionized calcium represents the physiologically active fraction of calcium and is critical in coagulation, myocardial contractility, and vascular tone. It is influenced by pH: acidosis increases ionized calcium due to reduced protein binding, while alkalosis reduces it [21].

### Limitations

While this review provides a comprehensive synthesis of available evidence on ABG and electrolyte analysis in heart failure, several limitations should be acknowledged. First, the discussion is based primarily on observational studies, registry data, and retrospective analyses, which may be subject to selection bias and confounding. As such, the prognostic associations identified between ABG parameters and clinical outcomes do not imply causality. Second, most of the evidence pertains to acute heart failure and cardiogenic shock, with fewer robust data available for stable chronic HF populations. Third, the variability in ABG measurement techniques, timing of sampling, and institutional protocols may limit the generalizability of findings across different clinical settings. Furthermore, this narrative review did not employ a systematic methodology for study selection, which may introduce publication bias. Although key references from major guidelines and high-quality studies were prioritized, the absence of formal quality assessment or meta-analytic techniques may limit the reproducibility of conclusions.

## 5. Conclusions and Clinical Application

ABG analysis has emerged as a vital tool in the management of HF, enabling rapid assessment of acid-base balance, respiratory efficiency, tissue perfusion, and electrolyte status at the bedside. In both acute and chronic HF, ABG parameters such as lactate, arterial pH, PaCO_2_, PaO_2_, and bicarbonate concentrations yield essential prognostic information and offer a window into the complex interplay between cardiac, pulmonary, and renal systems [69].

The integration of ABG and electrolyte monitoring in HF care not only facilitates early detection of decompensation but also supports tailored therapeutic decision-making, including the initiation of inotropic support, adjustment of diuretic regimens, or escalation to respiratory or circulatory support.

The interaction between electrolytes, ABG patterns, and HF is modified by sex and age. Women with HF often present with lower body weight, higher prevalence of HFpEF, and more frequent use of thiazides for hypertension, all of which increase the risk of diuretic-induced hyponatremia and hypokalemia. In post-menopausal women, changes in sex hormone profile may further alter renal sodium handling and vasopressin sensitivity, predisposing to dilutional hyponatremia. By contrast, men more commonly present with HFrEF, larger ischemic burden, and more severe neurohormonal activation, which are associated with higher rates of hyperkalemia when RAAS-inhibiting therapies are optimized.

Age also acts as a major effect modifier. Older patients have reduced GFR, diminished renal acid excretion, and lower respiratory reserve, which favor chronic hypercapnia and chronic metabolic compensation. Consequently, the same diuretic dose may produce more pronounced hypochloremia and metabolic alkalosis in an elderly patient than in a younger adult, and superimposed lactic acidosis may be partially masked on ABG. In younger patients, preserved respiratory drive and renal function often allow a more “pure” respiratory alkalosis or metabolic acidosis pattern in acute decompensation, with fewer mixed disorders.

Beyond sex and age, several comorbidities systematically influence electrolyte and ABG profiles in HF, including chronic kidney disease, COPD/OSA, obesity, and diabetes, as well as therapies such as SGLT2 inhibitors, MRAs, and acetazolamide. A careful interpretation of ABGs in HF should therefore integrate patient age, sex, comorbidity profile, and current pharmacotherapy to avoid misattributing an acid–base disturbance solely to HF decompensation.

As demonstrated in this review, hyperlactatemia and acidemia are robust markers of impaired perfusion and predict adverse outcomes [70, 71], while derangements in sodium and potassium are associated with increased morbidity and mortality [72, 73, 74, 75].

Moreover, serial ABG measurements provide dynamic feedback on treatment efficacy and can guide ongoing hemodynamic optimization. The ability to rapidly detect mixed or evolving acid-base disturbances, particularly in critically ill patients, underscores the value of ABG as a real-time, physiologic monitor.

Looking ahead, the expanding capabilities of point-of-care testing and integrated clinical decision support systems may enhance the precision and utility of ABG analysis in HF management. Future research should aim to standardize ABG-guided risk stratification algorithms and explore its integration with biomarker-based and imaging modalities to refine prognostic models.

Miniaturized devices capable of measuring pH, PaO_2_, PaCO_2_, lactate, and key electrolytes at the bedside, in ambulatory clinics, or even in home-based care models could allow earlier detection of decompensation and more agile titration of diuretics, RAAS inhibitors, and SGLT2 inhibitors. When combined with non-invasive hemodynamic monitoring and weight, blood pressure, and symptom data, serial ABG-like measurements may contribute to a richer “digital phenotype” of HF that captures both congestion and perfusion status.

In parallel, machine learning–based risk models offer an opportunity to integrate ABG parameters, electrolytes, biomarkers, imaging, and comorbidity profiles into dynamic prognostic and decision-support tools. Such models could support clinicians in identifying patients at high risk of deterioration shortly after presentation, refining the selection of candidates for intensive monitoring, non-invasive or invasive ventilation, or early mechanical circulatory support. However, these approaches require rigorous prospective validation, attention to data quality and calibration, and transparent, interpretable algorithms to ensure that they augment rather than complicate bedside decision-making.

In summary, ABG analysis, when interpreted in the appropriate clinical and pathophysiological context, represents a cornerstone of personalized, physiology-guided care in heart failure. By integrating information on acid-base status, oxygenation, ventilation, tissue perfusion, and electrolyte balance, ABG supports earlier recognition of decompensation, more precise risk stratification, and timely escalation or de-escalation of therapy. Emphasizing its systematic use in routine practice, and exploring its incorporation into structured risk scores and digital decision-support tools, may help reduce treatment delays and ultimately improve outcomes for patients across the spectrum of HF.
